# Distributed Identity Authentication with Lenstra–Lenstra–Lovász Algorithm–Ciphertext Policy Attribute-Based Encryption from Lattices: An Efficient Approach Based on Ring Learning with Errors Problem

**DOI:** 10.3390/e26090729

**Published:** 2024-08-27

**Authors:** Qi Yuan, Hao Yuan, Jing Zhao, Meitong Zhou, Yue Shao, Yanchun Wang, Shuo Zhao

**Affiliations:** 1 Faculty of Communication and Electronic Engineering, Qiqihar University, Qiqihar 161000, China; foreveryuanqi@126.com (Q.Y.); jzhao990809@gmail.com (J.Z.); zhoumeitong0806@163.com (M.Z.); 01480@qqhru.edu.cn (Y.W.); 2 State Grid Information Communication Branch, Beijing 100032, China; shaoyue126069@163.com; 3 Network Information Center, Qiqihar University, Qiqihar 161006, China; 01513@qqhru.edu.cn

**Keywords:** lattice cryptography, encryption of policy attributes, identity authentication, Lenstra–Lenstra–Lovász lattice reduction algorithm

## Abstract

In recent years, research on attribute-based encryption (ABE) has expanded into the quantum domain. Because a traditional single authority can cause the potential single point of failure, an improved lattice-based quantum-resistant identity authentication and policy attribute encryption scheme is proposed, in which the generation of random values is optimized by adjusting parameters in the Gaussian sampling algorithm to improve overall performance. Additionally, in the key generation phase, attributes are processed according to their shared nature, which reduces the computational overhead of the authorization authority. In the decryption phase, the basis transformation of the Lenstra–Lenstra–Lovász (LLL) lattice reduction algorithm is utilized to rapidly convert shared matrices into the shortest vector form, which can reduce the computational cost of linear space checks. The experimental results demonstrate that the proposed method not only improves efficiency but also enhances security compared with related schemes.

## 1. Introduction

With the continuous development of quantum computing technology, traditional encryption algorithms face unprecedented challenges. In this context, lattice-based cryptography has emerged as a promising choice to combat quantum attacks. Lattice-based cryptography was first proposed by Ajtai [1], who constructed an unbreakable cryptographic system using computationally hard lattice problems. This innovative solution not only lays the foundation for the development of post-quantum cryptography but also attracts widespread attention for its unique contributions to the field of cryptography. Therefore, lattice-based cryptography is regarded as an important milestone in the development of post-quantum cryptography.

In the field of cryptography, ciphertext policy attribute-based encryption (CP-ABE) and identity authentication technologies play a crucial role in information security and user identity verification. Considering the security threats during the process of data exchange in Internet of Thing (IoT), such as adversaries impersonating users to access data stored on servers or devices, receiving incomplete or tampered data, etc., research on post-quantum-based identity authentication and access control has become particularly urgent. In this context, the verification key protocol [2] based on the Ring Learning With Errors (RLWEs) assumption emerges as an important security service. Its main objective is to establish a session key after mutual authentication between users and accessed servers, which can ensure secure communication between users and servers. It provides a forward-looking solution for identity authentication and data access control issues in IoT environments, and it lays the foundation for more secure and reliable IoT communication.

CP-ABE technology has the dual advantages of fine-grained access control and data protection; thus, it attracts widespread attention. In recent years, the lattice-based [3] attribute encryption approach has become an important research direction in CP-ABE. Moreover, with in-depth research on the RLWEs problem, significant progress has been made in this direction, and lattice-based CP-ABE schemes have been successfully introduced into both centralized and decentralized systems. Continuous optimizations of the RLWEs problem have further enhanced the performance in implementing flexible access policies. For instance, in [4], the private keys are distributed by the decentralized multi-authority to improve efficiency, which is more suitable for distributed storage environments. This development gives the lattice-based CP-ABE schemes a unique advantage in the diverse and complex access control requirements of modern network environments.

In this paper, we made improvements based on reference [5], in which different third-party authorities and flexibility during communication are considered. Specifically, our improvements are mainly described in the following aspects:

- In the Gaussian sampling algorithm, two parameters are set for the standard deviation of the Gaussian distribution, which can concentrate the generated random values around the mean and ensures a wider Gaussian curve within a certain range of fluctuations. This results in a broader distribution of random values and improves overall performance.
- The RTrapGen algorithm handles shared and non-shared attributes differently. To enhance the efficiency of key generation, we first identify and categorize the hierarchical relationships of identities during initialization. Subsequently, in the AASetup phase, different sets of attributes are formed based on relationships, thereby reducing the computational overhead of the authorization authority. These optimization measures contribute to improving the performance of the algorithm.
- In the decryption phase, traditional Gaussian elimination is replaced to solve a set of scalar problems to verify the decryption result. A basis transformation is applied to the shared matrices in the Linear Secret Sharing Scheme (LSSS), and the shared matrices are rapidly converted into a collection of shortest vectors, thereby reducing the computational cost of linear space checks.

## 2. Related Work

So far, researchers have been continuously exploring and enhancing the security of CP-ABE schemes. These efforts aim to ensure that encryption systems effectively protect data from unauthorized access and disclosure. Zhang et al. [6] proposed an improved scheme for cloud computing CP-ABE, which closely associates access control policies with data to achieve fine-grained access control. The scheme also considers potential attacks through system information leakage rather than directly attacking the encryption algorithm, so it effectively maintains data security. Traditional cryptography is quite mature in resisting attacks, but further research on lattice-based cryptography is considered against quantum computers. Huang et al. [7] proposed a lattice-based group authentication scheme to resist various attacks. The scheme can implement group authentication where administrators can select any user to create the authentication process after confirming the total number of users. It indicates promising applications in the IoT domain. Sedat Akleylek et al. [8] proposed a new lattice-based IoT authentication scheme based on the ISIS problem, which can ensure system reliability against quantum attacks and meets zero-knowledge properties to protect privacy during authentication. It also defends against various attacks such as man-in-the-middle, simulation, and replay, while optimizing efficiency; therefore, it is suitable for RFID systems in the IoT. Through continuous security optimization, system protection can be effectively maintained while computational overhead is reduced. Fu et al. [9] proposed an offline/online lattice-based CP-ABE scheme, which can reduce the computational burden of mobile devices in two phases. With the RLWEs assumption, it enhances security against quantum computing attacks. And it is suitable for resource-constrained devices and has long-term security.

A gradual improvement has been achieved in lattice-based attribute strategies. However, challenges remain in lightweight and flexible encryption. Zhao et al. [10] proposed a revocable lattice attribute-based encryption scheme based on the RLWEs problem that can support attribute revocation and flexibly update user permissions to adapt to changing demands. Security proof is crucial for encryption schemes. In this scheme, security threats are discussed, such as collusion attacks [11,12,13], and corresponding solutions are proposed. This security analysis ensure that the schemes are not compromised by potential threats in practical applications. Further, researchers use the authentication scheme based on lattice in different application scenarios. Ali Shahidinejad et al. [14] presented a decentralized authentication and key exchange protocol for device-to-device communication in IoT, in which lattice-based encryption is used to resist quantum attacks and edge computing is introduced to reduce device computational overhead as well as improve system efficiency. This authentication scheme is applicable in smart homes [15], smart agriculture [16], and healthcare [17], which provides inter-domain authentication support.

Pithwi et al. [5] addressed a lattice-based quantum-resistant distributed identity authentication and policy attribute encryption scheme that can ensure the balance between security and lightweight encryption. This scheme uses ring variant trapdoors for lattice-based cryptography, which is suitable for distributed environments due to supporting the distributed settings. In the key generation and decryption phases, Shamir threshold secret sharing and Lagrange interpolation are employed for private key partitioning and recovery. Furthermore, Gaussian preimage sampling on lattice L is utilized for efficiency improvement. We find that a further improvement can be achieved based on [5]; for example, more stable parameters are found by the standard deviation of the distribution in Gaussian sampling algorithms to obtain a wider distribution of random values. Additionally, in the RTrapGen algorithm, the shared and the non-shared attribute sets are distinguished to facilitate computation. In the AASetup phase, different attribute sets are formed based on the shared and the non-shared attributes, which reduces the computational overhead of authorization authorities and indirectly enhances the efficiency of the key generation phase (KenGen). These optimization measures contribute to improving the performance of the algorithm. In the decryption phase, classical Gaussian elimination is replaced to address a set of scalar problems, which can determine the success of decryption. To achieve this improvement, a basis transformation is applied to the shared matrix F in the LSSS, which can be rapidly converted into a set of shortest vectors. Thereby, it can reduce the computational cost of linear space checks.

## 3. Preliminaries

In post-quantum cryptography, the mathematical structure of lattices has significant advantages in resisting quantum computing attacks based on lattice structures; the difficulty of the RLWEs problem is discussed in this paper. The RLWEs problem is established on a ring, and its security relies on the relationship between indistinct polynomials and random errors. Lattice-based RLWE algorithms provide an effective means against quantum attacks by leveraging the characteristics of rings and errors. This approach is widely applied in practical scenarios such as distributed identity authentication and attribute-based encryption schemes to ensure secure communication and data protection. In this section, we discuss the mathematical foundation and structure of lattice-based RLWE problems in the quantum domain, as well as the techniques adopted in distributed identity authentication and attribute-based encryption schemes.

### 3.1. Lattices

**Definition** **1.** *Taking into account an n-dimensional lattice, each base vector $b_{i}$* *comprises n* *real number vectors, denoted by $b_{i}=\left\{b_{i1},b_{i2},\ldots ,b_{in}\right\}$, where $b_{ij}\left(i,j=1,\ldots ,n\right)$* *represents the j-th component of the i-th base vector. Thus, the basis of L* *becomes a set $D=\{b_{1},b_{2},\ldots ,b_{n}\}$, where $b_{i}\in R^{n}$* *and R* *are polynomial rings. Then, the lattice L* *can be represented as $L\left(D\right)=\left\{\sum_{i=1}^{n}X_{i}\cdot b_{i}|X_{i}\in Z\right\}$. Furthermore, for the P-norm $l_{P\;\mathrm{norm}}$* *on lattice vector X, it is defined as ${\left\|X\right\|}_{P}={\left({\sum_{i=1}^{n}\left|X_{i}\right|}^{P}\right)}^{1/P}$, where $X_{i}$* *is the ith component of vector X*.

### 3.2. RLWEs

**Definition** **2.** *Given a polynomial ring $R_{q}=\frac{Z_{q}\left[x\right]}{\langle x^{f}+1\rangle }$, a secret polynomial S, and an error polynomial $\bar{e}$, we define the following sampling process: randomly selecting $a\in R_{q}$. Calculate $b=a\cdot S+\bar{e}$, where all calculations are performed on the ring of module q. The opponent obtains a set of sample pairs (a,b), generated from the above process or a completely random distribution. Specifically, for example, the truly random oracle O* *is described as follow. The generated samples for (a,b)* *are entirely random, where polynomial a and polynomial b are randomly selected from $R_{q}$* *with a uniform distribution. The RLWEs oracle $O_{s}$* *is described as follow. In the generated sample pairs (a,b), where the polynomial a* *is randomly selected from a uniform distribution on $R_{q}$, S* *is described as a fixed secret polynomial, and the noise $\bar{e}$* *is extracted from a discrete Gaussian distribution. The decision RLWEs assume that the opponent cannot significantly distinguish between samples from the RLWEs oracle $O_{s}$* *and samples from the genuinely random oracle O* *in polynomial time. Therefore, the difficulty of the decision RLWEs problem lies in the difficulty that the opponent recovers the secret polynomial S* *from a given sample, even if the opponent knows the process of generating the sample [18].*

### 3.3. Gaussian Sampling

Discrete Gaussian sampling and Gaussian inversion sampling are widely applied in the fields of computer science and cryptography. The former refers to the process of generating random samples from a discrete Gaussian distribution, while the latter refers to the process of generating random samples from a standard normal distribution (Gaussian distribution with mean 0 and variance 1). We briefly introduce these two sampling methods.

**Definition** **3** **(Discrete Gaussian Sampling).** *A Gaussian function with center distance $c\left(c\in R^{n}\right)$* *and density function σ(∀σ>0) is denoted as $\rho_{\sigma ,c}(x)=exp\left(-\pi \frac{{\left\|x-c\right\|}^{2}}{\sigma^{2}}\right)$. Gaussian distribution based on an n-dimensional lattice L(D)* *is defined as $\rho_{\sigma ,c\left(L\left(D\right)\right)}=\sum_{x\in L\left(D\right)}\rho_{\sigma ,c}\left(x\right)$, and a discrete Gaussian distribution [5] is defined as ∀y∈L(D), $D_{L\left(D\right),\sigma ,c\left(y\right)}=\frac{\rho_{\sigma ,c}\left(y\right)}{\rho_{\sigma ,c}\left(L\left(D\right)\right)}$. In this paper, the Gaussian function value is calculated at each point on the lattice, and all the values are summed to obtain the total. Then, the Gaussian function value at each point is divided by this total. As a result, the sum of the normalized Gaussian function values will be 1. This normalization ensures that the integral of the function can be 1 over the entire lattice, which meets the properties of a probability distribution*.

**Definition** **4** **(Gaussian preimage sampling).** *As shown in Algorithm 1 below, $RSamplePre\left(A,{T^{\prime }}_{A},v,\sigma ,\sigma_{s}\right)\to \left(M\right)$. Given input vector $A\in R_{q}^{1\times m}$, the trap ${T^{\prime }}_{A}=\left(r^{\prime },e^{\prime }\right)$, a vector $v\in R_{q}$, and the parameters $\sigma ,\sigma_{s}\left(\sigma ,\sigma_{s}>0\right)$, an interference vector $l^{\prime }\in R_{q}^{m}$* *is generated, while the vector $Y=R_{q}^{k}$* *is computed by $J^{T}\cdot Y=v-A\cdot l^{\prime }$, where a vector $J^{T}=\{J_{1},J_{2},\ldots ,J_{K}\}$, $J_{i}=2^{i-1}$* *for all i∈[k], $k=\left\lfloor \log_{b}q+1\right\rfloor$. The vector $M={\left[{l^{\prime }}_{1}+e^{\prime }\cdot Y,{l^{\prime }}_{2}+e^{\prime }\cdot Y,{l^{\prime }}_{3}+Y_{1},\ldots ,{l^{\prime }}_{m}+Y_{k}\right]}^{T}\in R_{q}^{m}$* *is obtained as the output and M* *is sampled from $D_{\wedge q\left(A\right),\sigma_{s}}$* *when A·M=v* *is true* [11].

**Algorithm 1:** RSamplePre **Input:** $A,{T^{\prime }}_{A},v,\sigma ,\sigma_{s}$
 **Output:** 
M


$1\;\mathrm{generate}\;l^{\prime }\left(l^{\prime }\in R_{q}^{m}\right)$



$2\;\mathrm{generate}\;Y\left(Y\in R_{q}^{k}\right)$

3 for i=0,1,…,m−1 **do**4 if i==0 **then**

$5\;\;\mathrm{compute}M_{0}={l^{\prime }}_{1}+e^{\prime }\cdot Y$

6 else if i==1 **then**

$7\;\;\mathrm{compute}\;M_{1}={l^{\prime }}_{2}+r^{\prime }\cdot Y$

8 else if 2≤i≤k+1 **then**

$9\;\;\mathrm{compute}\;M\left[i\right]={l^{\prime }}_{i+1}+Y\left[i-1\right]$

10 **else**

$11\;\;\mathrm{compute}\;M\left[i\right]={l^{\prime }}_{i+1}$

12 **end if**13 **end for**

14 verify if A·M=v

15 **if true then**
16 **return M**17 **else**18 **return Error**19 **end if**

### 3.4. Trapdoor

**Definition** **5.** *As shown in Algorithm 2, $Trapdoor\left(q,f,k,\sigma \right)\to \left(A,T_{A}^{\prime }\right)$. Consider a vector $J^{T}\leftarrow \left[J_{1},J_{2},J_{3},\ldots ,J_{k}\right]$, where $J_{i}=2^{i-1}\left(i\in \left[k\right]\right)$. Assume q=q(λ)* *is prime. There are $f=f\left(\lambda \right)\in Z^{+}$, σ=σ(λ), $k=\left\lceil \log_{b}q+1\right\rceil$, where λ* *is a security parameter and b* *is the cardinality of vector $J^{T}$, while b* *is at least 2. Give an output vector A* *and a trapdoor ${T^{\prime }}_{A}$, where the size of A is m=k+2. Define $A=\left[1,a,J_{1}-\left[a\cdot {r^{\prime }}_{1}+{e^{\prime }}_{1}\right],\ldots ,J_{k}-\left[a\cdot {r^{\prime }}_{k}+{e^{\prime }}_{k}\right]\right]\in R_{q}^{1\times m}$* *and ${T^{\prime }}_{A}=\left(r^{\prime },e^{\prime }\right)$, where $a\in R_{q}$. The security of this algorithm relies on the RLWEs assumption, where ${T^{\prime }}_{A}$* *is secret, and $\left(r^{\prime },e^{\prime }\right)\in R_{q}^{k}\times R_{q}^{k}$* *is generated by a Gaussian distribution $D_{R,\sigma }$ [12].*

**Algorithm 2:** Trapdoor Generation** Input:** 
q,f,k,σ
** Output:** 
$A,{T^{\prime }}_{A}$


$1\;\mathrm{Construct}\;J^{T}\leftarrow \left[1,2^{1},2^{2},\ldots ,2^{k-1}\right]$



$2\;\mathrm{generate}\;a\in R_{q}$



$3\;\mathrm{generate}\;\left(r^{\prime },e^{\prime }\right)\in R_{q}^{k}\times R_{q}^{k}$

$4\;\mathrm{Initialize}\;A\in R_{q}^{1\times m}$ 
**with** 
m=k+2


$5\;\mathrm{Set}\;A_{1}=1,A_{2}=a$

6 **for i = 1,2,...,k do**

$7\;\;A_{i+2}=J_{i}-\left(a\cdot {r^{\prime }}_{i}+{e^{\prime }}_{i}\right)$

8 **end for**

$9\;\mathrm{return}\;A,{T^{\prime }}_{A}$



### 3.5. Security Assumptions

In the field of cryptography, we assume that the security model consists of a series of games between a Probabilistic Polynomial Time (PPT) adversary A and a challenger C. As the adversary, its attacks include launching traditional number-theoretic attacks and quantum attacks against RLWE-based systems simultaneously. These attacks include replay attacks, man-in-the-middle attacks, temporary secret leakage attacks, signal leakage attacks, and so on. The security model described in this paper is considered secure against selectively ciphertext attacks (sCPA).

During the initialization phase, adversary A will attempt to attack and disrupt the access structure or permissions and declare two internal challenges: access structure challenge and a set of compromised permissions $J_{c}$. These challenges are then sent to C. Challenger C executes Setup and AASetup algorithms to generate public parameters and the public–private key pairs corresponding to each compromised institution in the $J_{c}$ list. C forwards the generated parameters to adversary A.

Phase 1: Adversary A attempts to obtain private key information for compromised permissions. Adversary A generates $\left(uid,S_{uid}\right)$ and sends it to C. Meanwhile, A frequently sends queries for private key generation to C. Here, $S_{uid}$ represents the attribute set of user uid. T represents the attribute set associated with the compromised permissions. Since $\left|S_{uid}\cap T\right|$ does not satisfy the challenge access structure $W^{\prime }$, the key generation algorithm keyGen is executed by C, and C forwards the generated private key to adversary A.

Challenge: Adversary A randomly selects two messages, $\phi_{1}$ and $\phi_{2}$, which can be seen as choices of plaintext to be encrypted. These two messages are sent to C, which simulates a step of requesting encryption for C. C selects a value α from {0,1} that represents the message encrypted by challenger C. According to the challenge access structure $W^{\prime }$, challenger C encrypts message $\phi_{\alpha }$ using the selected α. C sends the generated ciphertext ct to adversary A.

Phase 2: In this stage, adversary A frequently requests key queries.

Conjecture: Adversary A engages in a game where A guesses $\alpha^{\prime }\in \left\{0,1\right\}$ about variable α. If $\alpha^{\prime }=\alpha$, adversary A wins the game. The probability of winning is defined as the advantage of A, namely Adv(A), where $A=\mathrm{Pr}\left[a^{\prime }=a\right]-\frac{1}{2}$. If $a^{\prime }=a$ is true, it denotes that the guess $\alpha^{\prime }=0$ is correct. On the contrary, the guess $\alpha^{\prime }=1$ is true. In summary, it is defined as Equation (1).

(1) $$\begin{array}{ll} Adv\left(A\right)=\left|\mathrm{Pr}\left[\alpha^{\prime }=\alpha \right]-\frac{1}{2}\right| & =\left|\begin{array}{l} \mathrm{Pr}\left[\alpha^{\prime }=\alpha |\alpha =0\right]\mathrm{Pr}\left[\alpha =0\right]\\ +\mathrm{Pr}\left[\alpha^{\prime }=\alpha |\alpha =1\right]\mathrm{Pr}\left[\alpha =1\right]-\frac{1}{2} \end{array}\right|\\ & =\left|\left(A+\frac{1}{2}\right)\frac{1}{2}+\frac{1}{2}\times \frac{1}{2}-\frac{1}{2}\right|\\ & =\frac{A}{2} \end{array}$$

Therefore, this assumption is not feasible, but it has an undeniable advantage in solving the above conjecture. Our proposed lattice-based CP-ABE scheme with multiple authorities is secure in the sCPA model. Specifically, if there exists an adversary A who can successfully break IND-sCPA security (i.e., with a non-negligible success probability A(A>0)), we can deduce that another adversary B can solve the RLWEs problem with a corresponding advantage (at least $\frac{A}{2}$). It demonstrates that the security of the CP-ABE scheme is closely related to the difficulty of the RLWEs problem.

### 3.6. Formal Definition for CP-ABE

Setup. The implicit security parameter is given to the setup algorithm as the input. It outputs public parameters $paras=\left\{q,f,k,\sigma ,\sigma_{s},u\right\}$.

AASetup $\left(paras,{\chi^{\prime }}_{\theta },P_{\theta }\right)$. Each authorization authority $AA_{\theta }$ runs RTrapGen by paras, outputs a key pair $\left(A_{\theta },T_{A_{\theta }}^{\prime }\right)$, selects an attribute set ${\chi^{\prime }}_{\theta }$, chooses a polynomial $P_{\theta }$, and finally generates a public key $AP{K^{\prime }}_{\theta }$ and private key $AS{K^{\prime }}_{\theta }$.

KenGen $\left({\chi^{\prime }}_{\theta },A_{\theta },T_{A_{\theta }}^{\prime },\Delta_{\theta },\sigma ,\sigma_{s},SK_{uid}\right)$. The KenGen algorithm takes the attribute set ${\chi^{\prime }}_{\theta }$ and the public parameters $\left(A_{\theta },T_{A_{\theta }}^{\prime },\Delta_{\theta },\sigma ,\sigma_{s}\right)$ from the Gaussian image sampling algorithm as inputs and outputs the private key $SK_{uid}$.

Encryption $\left(AP{K^{\prime }}_{\theta },W,M_{ess},F,\Sigma ,E_{n}\left(M\right)\right)$. The public key $AP{K^{\prime }}_{\theta }$, access structure W, plain text $M_{ess}$, matrix F, and attribute vector Σ are all inputted to the KenGen algorithm. The algorithm will encrypt $M_{ess}$ and produce a ciphertext $E_{n}\left(M\right)$ so that only a user that possesses a set of attributes that satisfy the access structure can decrypt the message.

Decryption $\left(F,E_{n}\left(M\right),SK_{uid}\right)$. The matrix F, ciphertext $E_{n}\left(M\right)$, and the private key $SK_{uid}$, which is regarded as a private key for a set ${\chi^{\prime }}_{\theta }$ of attributes, are described as the inputs of the decryption algorithm. The decrypted ciphertext $M_{ess}$ is obtained in the condition of satisfying different access structures.

## 4. Lattice-Based Multi-Authority CP-ABE

In this section, security assumption is discussed, and the multi-authority CP-ABE proposal based on lattice is implemented. The meanings of the symbols in the scheme are shown in Table 1.

### 4.1. System Model

The description of the system model is as follows: Firstly, the trusted key generation center KGC generates public parameters, permissions, and unique identities corresponding to legitimate users by executing the Setup and AASetup stages. The access policy is set based on the general attributes of the data owner. Secondly, during the encryption phase, the ciphertext is uploaded to the cloud server. Data users download ciphertext from cloud servers. For this purpose, data users must send requests to various institutions to publish their private keys. Data users can decrypt the ciphertext only when their private keys meet the access policy.

### 4.2. Scheme Design

**Figure 1:**
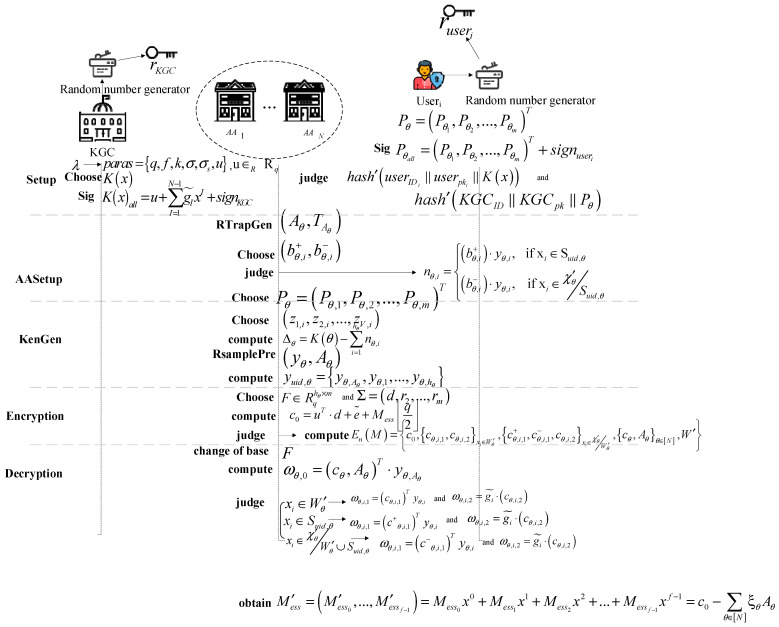
The system model of this scheme.

The overall scheme, as depicted in Figure 1, comprises five stages: initialization setup (Setup), attribute authority setup (AASetup), key generation (KenGen), encryption, and decryption. Then, a detailed description of each stage is provided as follows.

#### 4.2.1. Setup

A trusted key generation center (KGC) inputs security parameter λ during the initialization phase. A polynomial $u\in_{R}\;R_{q}$ is selected, and the public parameter $paras=\left\{q,f,k,\sigma ,\sigma_{s},u\right\}$ is outputted. Assuming that the number of authorization agencies is represented as N, the set of authorization agencies is described as $\left\{AA_{1},AA_{2},\ldots ,AA_{N}\right\}$, where I∈{1,2,…,N−1}. KGC uniformly selects the polynomial K(x) of degree *N* − 1, where $K\left(x\right)=u+\sum_{I=1}^{N-1}\tilde{g_{I}}x^{I}$, $\tilde{g_{I}}\in R_{q}$. During the process of selecting polynomial K(x), KGC signs K(x) named as $sign_{k\left(x\right)}$ and obtains $K{\left(x\right)}_{all}=u+\sum_{I=1}^{N-1}\tilde{g_{I}}x^{I}∥sign_{KGC}$. KGC obtains temporary private key $r_{KGC}$ through a random number generator and then calculates temporary public key $KGC_{pk}=r_{KGC}\cdot P\mathrm{mod}p$. And the signature of KGC is generated as $sign_{KGC}=hash\left(KGC_{ID}∥KGC_{pk}∥P_{\theta }\right)$. Users obtain the temporary private key $r_{user_{i}}$ through a random number generator and then calculate temporary public key $user_{pk_{i}}=r_{user_{i}}\cdot P\mathrm{mod}p$. When users choose $P_{\theta }={\left(P_{\theta_{1}},P_{\theta_{2}},\ldots ,P_{\theta_{m}}\right)}^{T}$, where θ is a weight of permission, they digitally sign the relevant information $P_{\theta_{all}}={\left(P_{\theta_{1}},P_{\theta_{2}},\ldots ,P_{\theta_{m}}\right)}^{T}∥sign_{user_{i}}$, where $sign_{user_{i}}=hash\left(user_{ID_{i}}∥user_{pk_{i}}∥K\left(x\right)\right)$. Before transmitting K(x) and $P_{\theta }$ to the corresponding authority $AA_{\theta }$, the temporary public key $KGC_{pk}$ and $user_{pk_{i}}$ are transformed through a secure channel.

#### 4.2.2. AASetup

$AA_{\theta }$ first runs the RTrapGen algorithm that can output a pair of parameters $\left(A_{\theta },T_{A_{\theta }}\right)$, where $A_{\theta }\in R_{q}^{1\times m}$, $T_{A_{\theta }}=\left(r_{\theta },e_{\theta }\right)$ and $r_{\theta }$, $e_{\theta }\in R_{q}^{k}$. If ${\chi^{\prime }}_{\theta }=\left\{x_{1},x_{2},\ldots ,x_{h_{\theta }}\right\}$ represents the attribute set managed by $AA_{\theta }$, for each attribute $x_{i}\in {\chi^{\prime }}_{\theta }$, $\left(b_{\theta ,i}^{+},b_{\theta ,i}^{-}\right)$ is uniformly selected from $R_{q}^{1\times m}\times R_{q}^{1\times m}$ at random. The affiliation relationship of the attribute (shared attribute or non-shared attribute set) is determined, i.e., $x_{i}\in {\chi^{\prime }}_{\theta }$. The determination formula is the following Equation (2).

(2) nθ,i={(bθ,i+)·yθ,i, if xi∈Suid,θ (bθ,i−)·yθ,i, if xi∈χ′θSuid,θ

The three cases are obtained as follows: (a) the elements ${\chi^{\prime }}_{\theta }$ in the attribute set $x_{i}$ belong to the access structure $W_{\theta }^{+}$, i.e., $x_{i}\in W_{\theta }^{+}$. (b) The elements ${\chi^{\prime }}_{\theta }$ in the attribute set $x_{i}$ do not belong to the access structure $W_{\theta }^{+}$, i.e., $x_{i}\in W_{\theta }^{-}$. (c) The elements ${\chi^{\prime }}_{\theta }$ in the attribute set $x_{i}$ are not allowed to access structure ${W^{\prime }}_{\theta }$. Then, $AA_{\theta }$ selects $P_{\theta }={\left(P_{\theta ,1},P_{\theta ,2},\ldots ,P_{\theta ,m}\right)}^{T}$. For each Π∈[m], there is $P_{\theta ,\Pi }=\sum_{U=0}^{f-1}P_{\theta ,\Pi ,U}\left(z_{1,i},z_{2,i},\ldots ,z_{V,i}\right)x^{U}$, where $\left(z_{1,i},z_{2,i},\ldots ,z_{V,i}\right)\in {\left(Z_{q}\right)}^{V}$. It is worth noting that for all θ∈[N], Π∈[m], U∈{0,…,f−1}, and $P_{\theta ,\Pi ,U}$, they are linearly distributed on $\left(z_{1,i},z_{2,i},\ldots ,z_{V,i}\right)$. Thus, the public key $AP{K^{\prime }}_{\theta }$ and private key $AS{K^{\prime }}_{\theta }$ for $AA_{\theta }$ are derived, respectively, as $\left\{A_{\theta },{\left(b_{\theta ,i}^{+},b_{\theta ,i}^{-}\right)}_{i\in \left[h_{\theta }\right]}\right\}$ and $\left\{T_{A_{\theta }},P_{\theta }\right\}$.

#### 4.2.3. KenGen

For each attribute $x_{i}\in {\chi^{\prime }}_{\theta }$ of authority $AA_{\theta }$, where $i\in \left[h_{\theta }\right]$, the authority randomly selects vectors $\left(z_{1,i},z_{2,i},\ldots ,z_{V,i}\right)$ from ${\left(Z_{q}\right)}^{V}$. Then, by computing polynomial $P_{\theta }$, it obtains vector $y_{\theta ,i}={\left(P_{\theta }\right)}_{\left(z_{1,i},z_{2,i},\ldots ,z_{V,i}\right)}$. This process is aimed at generating polynomial values associated with the attributes of authority $AA_{\theta }$. For $y_{\theta ,i}\in R_{q}^{m}$, ∀θ∈[N], $i\in \left[h_{\theta }\right]$, the authority $AA_{\theta }$ calculates the difference vector $\Delta_{\theta }=K\left(\theta \right)-\sum_{i=1}^{h_{\theta }}n_{\theta ,i}$ based on the judgments in AASetup and runs the RSamplePre algorithm to obtain $\left(y_{\theta },A_{\theta }\right)$. This step is intended to generate encrypted vectors of user attribute values based on the specified distribution to enhance the security of the keys. As a result, the authority $AA_{\theta }$ includes the output vector $\left(y_{\theta },A_{\theta }\right)$ as part of the private key, i.e., $SK_{uid}=\left\{y_{uid};\theta \in \left[N\right]\right\}$, where $y_{uid,\theta }=\left\{y_{\theta ,A_{\theta }},y_{\theta ,1},\ldots ,y_{\theta ,h_{\theta }}\right\}$.

#### 4.2.4. Encryption

The user receives public keys $APK_{\theta }$ provided by the authority $AA_{\theta }$, where *θ*(θ∈[N]) includes the access structure denoted as ${W^{\prime }}_{\theta }$. And attribute assignments relate to each authority. The user constructs the overall access structure $W^{\prime }=\cup_{\theta \in \left[N\right]}{W^{\prime }}_{\theta }$. These attributes are merged when constructing the access structure, denoted as $W^{\prime }=\left(W_{\theta }^{+}\cup W_{\theta }^{-}\right)$. The plaintext message is represented as $M_{ess}=\left(M_{ess_{0}},M_{ess_{1}},\ldots ,M_{ess_{f-1}}\right)\in {\left\{0,1\right\}}^{f}$, in which it is expressed as a polynomial $M_{ess}\left(x\right)=M_{ess_{0}}x^{0}+M_{ess_{1}}x^{1}+M_{ess_{2}}x^{2}+\ldots +M_{ess_{f-1}}x^{f-1}$. The user selects an attribute vector $F\in R_{q}^{h_{\theta }\times m}$ and $\Sigma =\left(d,r_{2},\ldots ,r_{m}\right)$, where $d\in R_{q}$ is a shared secret and $\left(r_{2},\ldots ,r_{m}\right)\in R_{q}$. Then, random error term $\tilde{e}$ is selected from the same Gaussian distribution $D_{R,\sigma }$. The ciphertext $c_{0}=u^{T}\cdot d+\tilde{e}+M_{ess}\left\lfloor \frac{q}{2}\right\rfloor$ is computed, where u is a constant, d is the shared secret, $M_{ess}\left\lfloor \frac{q}{2}\right\rfloor$ quantifies the plaintext, and q is a large prime. Random samples $\left(e_{\theta },A_{\theta }\right)\in R_{q}^{1\times m}$ from $D_{R,\sigma }$ are then encrypted to obtain $c_{\theta ,A_{\theta }}=A_{\theta }^{T}\cdot d+e_{\theta ,A_{\theta }}$ by $c_{0}$. There are three kinds of correspondence between the user and the authority’s attributes. Accordingly, three different encryption schemes are accomplished.

(1) The two samples $e_{\theta ,i,1}\in R_{q}^{1\times m}$ and $e_{\theta ,i,2}\in R_{q}^{m}$ constitute confusion factors $c_{\theta ,i,1}$ and $c_{\theta ,i,2}$ accordingly, where $c_{\theta ,i,1}=\left(b_{\theta ,i}^{+}\right)\cdot d+e_{\theta ,i,1}$ and $c_{\theta ,i,2}=\left(F_{i,1}\right)\cdot u^{T}\cdot d+\sum_{j=2}^{m}F_{i,j}\cdot r_{j}+e_{\theta ,i,2}$.
(2) The two samples $e_{\theta ,i,1}\in R_{q}^{1\times m}$ and $e_{\theta ,i,2}=\left(F_{i,1}\right)\cdot u^{T}\cdot d+\sum_{j=2}^{m}F_{i,j}\cdot r_{j}+e_{\theta ,i,2}$ constitute confusion factors $c_{\theta ,i,1}=\left(b_{\theta ,i}^{-}\right)\cdot d+e_{\theta ,i,1}$ and $c_{\theta ,i,2}=\left(F_{i,1}\right)\cdot u^{T}\cdot d+\sum_{j=2}^{m}F_{i,j}\cdot r_{j}+e_{\theta ,i,2}$ accordingly.
(3) Sample $e_{\theta ,i,1}^{+},e_{\theta ,i,1}^{-}\in D_{\theta ,\sigma }$ and sample $e_{\theta ,i,2}\in R_{q}^{m}$. For attribute $x_{i}\in W_{\theta }^{+}$, there is $c_{\theta ,i,1}^{+}=\left(b_{\theta ,i}^{+}\right)\cdot d+e_{\theta ,i,1}^{+}$, and attribute $x_{i}\in W_{\theta }^{-}$, there is $c_{\theta ,i,1}^{-}=\left(b_{\theta ,i}^{-}\right)\cdot d+e_{\theta ,i,1}^{-}$, as well as the standard item $c_{\theta ,i,2}=\left(F_{i,1}\right)\cdot u^{T}\cdot d+\sum_{j=2}^{m}F_{i,j}\cdot r_{j}+e_{\theta ,i,2}$; the form of the ciphertext is denoted as En(M)={c0,{cθ,i,1,cθ,i,2}xi∈W′θ,{cθ,i,1+,cθ,i,1−,cθ,i,2}xi∈χ′θW′θ,{cθ,Aθ}θ∈[N],W′}.

#### 4.2.5. Decryption

Taking a set of scalars $\tilde{g_{i}}\in \left\{0,1\right\},i\in \left[h_{\theta }\right]$, there is $\sum_{i=1}^{h_{\theta }}\tilde{g_{i}}\cdot F_{i}=\left(1,0,\ldots ,0\right)$, where $F_{i}$ represents the i-th row of matrix F. LLL is performed on the shared matrix F that replaced the LSSS of [5], where F is transformed into the set of shortest vectors (SVP) through basis transformation. If vector $\left(1,0,\ldots ,0\right)\in Span\langle F_{i},i\in \left[h_{\theta }\right]\rangle$ is true, where θ∈[N], it indicates that there exists a shortest vector after the LLL operation. And decryption will be successful. That is, each authority $AA_{\theta }$ calculates $\omega_{\theta ,0}={\left(c_{\theta },A_{\theta }\right)}^{T}\cdot y_{\theta ,A_{\theta }}$. According to the correspondence between user attributes and the attributes authorized by the authority,$\omega_{\theta ,i,1}$ and $\omega_{\theta ,i,2}\in R_{q}$ are computed as below:

(1) When $x_{i}\in {W^{\prime }}_{\theta }$ is true, compute $\omega_{\theta ,i,1}={\left(c_{\theta ,i,1}\right)}^{T}y_{\theta ,i}$, $\omega_{\theta ,i,2}=\tilde{g_{i}}\cdot \left(c_{\theta ,i,2}\right)$.
(2) For other $x_{i}\in S_{uid,\theta }$, compute $\omega_{\theta ,i,1}={\left({c^{+}}_{\theta ,i,1}\right)}^{T}y_{\theta ,i}$, $\omega_{\theta ,i,2}=\tilde{g_{i}}\cdot \left(c_{\theta ,i,2}\right)$.
(3) When xi∈χ′θW′θ∪Suid,θ is true, compute $\omega_{\theta ,i,1}={\left({c^{-}}_{\theta ,i,1}\right)}^{T}y_{\theta ,i}$, $\omega_{\theta ,i,2}=\tilde{g_{i}}\cdot \left(c_{\theta ,i,2}\right)$, $\omega_{\theta }=\omega_{\theta ,0}+\sum_{i=1}^{h_{\theta }}\left[\omega_{\theta ,i,1}+\omega_{\theta ,i,2}\right]\in R_{q}$.

Finally, the ciphertext parts are combined to obtain the final plaintext message ${M^{\prime }}_{ess}=\left({M^{\prime }}_{ess_{0}},\ldots ,{M^{\prime }}_{ess_{f-1}}\right)=M_{ess_{0}}x^{0}+M_{ess_{1}}x^{1}+M_{ess_{2}}x^{2}+\ldots +M_{ess_{f-1}}x^{f-1}=c_{0}-\sum_{\theta \in \left[N\right]}\xi_{\theta }A_{\theta }$, where $\xi_{\theta }=\frac{\Pi_{\theta \in \left[N\right],\theta \neq j}-\theta }{\Pi_{\theta \in \left[N\right],\theta \neq j}j-\theta }$ is a Lagrange polynomial. At this point, for each i∈{0,…,f−1}, it is necessary to determine whether the Lagrange interpolation polynomial $\left|{M^{\prime }}_{ess_{i}}\right|\overset{?}{<}\frac{q}{4}$ holds. If true, $M_{ess_{i}}=0$ is outputted; otherwise, $M_{ess_{i}}=1$ is outputted. This process converts the real value ${M^{\prime }}_{ess_{i}}$ obtained from interpolation into binary values. The basic idea of this method is as follows. A threshold $\frac{q}{4}$ is chosen. By comparing the threshold with the magnitude of $\left|{M^{\prime }}_{ess_{i}}\right|$, the binary information of $M_{ess_{i}}$ is determined that can effectively extract useful plaintext information from noise.

## 5. Secure Analysis and Performance Verification

In this section, the correctness is defined firstly from the view of probability of correctly recovered plaintext. Meanwhile, parameters are selected to ensure the correctness. Then, the secure proposed scheme is analyzed and the performance is verified.

### 5.1. Correctness and Parameter Selection

For all $AA_{\theta }$, the receiving party who held the attributes $SK_{uid}$ is considered secure if it satisfies the following two conditions: $\left\{ \begin{array}{l} SK_{uid,\theta }\cap W_{\theta }^{-}=\emptyset \\ SK_{uid,\theta }\cap W_{\theta }^{+}=W_{\theta }^{+} \end{array}\right.$. That is, the receiving party has sufficient attributes to meet the access policy of $AA_{\theta }$. Meanwhile, no excess attributes intersect with $W_{\theta }^{-}$. For ensuring the generation of a vector satisfying a specific distribution to enhance security, we perform the following calculation described as Equation (3).

(3) $$\begin{array}{l} \sum_{\theta \in \left[N\right]}\xi_{\theta }\omega_{\theta }\\ =\sum_{\theta \in \left[N\right]}\xi_{\theta }\left(\omega_{\theta ,0}+\sum_{i=1}^{h_{\theta }}\left[\omega_{\theta ,i,1}+\omega_{\theta ,i,2}\right]\right)\\ =\sum_{\theta \in \left[N\right]}\xi_{\theta }\left({\left(c_{\theta ,A_{\theta }}\right)}^{T}y_{\theta ,A_{\theta }}+\sum_{i=1}^{h_{\theta }}\left[{\left(c_{\theta ,i,1}\right)}^{T}y_{\theta ,i}+\tilde{g_{i}}\left(c_{\theta ,i,2}\right)\right]\right)\\ =\sum_{\theta \in \left[N\right]}\xi_{\theta }\left({\left(A_{\theta }^{T}d+e_{\theta ,A_{\theta }}\right)}^{T}y_{\theta ,A_{\theta }}+\sum_{i=1}^{h_{\theta }}\left[{\left(c_{\theta ,i,1}\right)}^{T}y_{\theta ,i}+\tilde{g_{i}}\left(c_{\theta ,i,2}\right)\right]\right)\\ =\sum_{\theta \in \left[N\right]}\xi_{\theta }\left(y_{\theta ,A_{\theta }}\left(A_{\theta }^{T}d\right)+y_{\theta ,A_{\theta }}e_{\theta ,A_{\theta }}+\sum_{i\in \left[h_{\theta }\right]}\langle n_{\theta ,i},d\rangle +\sum_{j\in \left[h_{\theta }\right]}e_{\theta ,j}\right)\\ =\sum_{\theta \in \left[N\right]}\xi_{\theta }\left(u_{\theta }d+y_{\theta ,A_{\theta }}e_{\theta ,A_{\theta }}+\sum_{j\in \left[h_{\theta }\right]}y_{\theta ,j}e_{\theta ,j}\right) \end{array}$$

In summary, the plaintext is computed as Equation (4), which can ensure the precision of the scheme.

(4) $$\begin{array}{ll} {M^{\prime }}_{ess} & =c_{0}-\sum_{\theta \in \left[N\right]}\xi_{\theta }\left(u_{\theta }d+y_{\theta ,A_{\theta }}e_{\theta ,A_{\theta }}+\sum_{j\in \left[h_{\theta }\right]}y_{\theta ,j}e_{\theta ,j}\right)\\ & \approx M_{\mathrm{ess}}\left\lfloor \frac{q}{2}\right\rfloor \end{array}$$

The error term $\left|\tilde{e}-\sum_{\theta \in \left[N\right]}\xi_{\theta }\left(u_{\theta }d+y_{\theta ,A_{\theta }}e_{\theta ,A_{\theta }}+y_{\theta ,1}e_{\theta ,1}+\ldots +y_{\theta ,h_{\theta }}e_{\theta ,h_{\theta }}\right)\right|<\frac{q}{4}$ must be constrained that can ensure the correct decryption. According to security constraints and parameter selection, the probability of the correct decryption depends on the norm of the private key generated by the Gaussian preimage sampling algorithm and the error term introduced during encryption. As stated in Section 4.1, they are set to 2 and 3, respectively.

Let the upper limit of $\left|e_{\theta ,A_{\theta }},e_{\theta ,1},\ldots ,e_{\theta ,h_{\theta }}\right|$ and $\left|y_{\theta ,A_{\theta }},y_{\theta ,1},\ldots ,y_{\theta ,h_{\theta }}\right|$ be $\Delta_{e}$ and $\Delta_{y}$, respectively. The central limit theorem estimates the noise factor $\left|y_{\theta ,A_{\theta }}e_{\theta ,A_{\theta }}+y_{\theta ,1}e_{\theta ,1}+\ldots +y_{\theta ,h_{\theta }}e_{\theta ,h_{\theta }}\right|$ as $\Delta =\Delta_{e}\Delta_{y}\sqrt{Nnm\left(h_{\theta }+1\right)}$, and parameters $\Delta_{e}=8\sigma$, $\Delta_{y}=8\sigma_{s}$ are set based on the literature [19]. Therefore, the correctness constraint is $q\geq 256\sigma \sigma_{s}\sqrt{Nnm\left(h_{\theta }+1\right)}$.

### 5.2. Security Analysis

The proposed scheme relies on assumptions made in the security model to analyze the mainstream attacks that may be faced in modern networks, traditional number theory cryptography, and quantum cryptography.

- Replay Attack: In the setup stage, KGC selects a polynomial K(x) when using random $\tilde{g_{I}}$ and conducts a random selection for both $\left({b^{+}}_{\theta ,i},{b^{-}}_{\theta ,i}\right)$ and $P_{\theta }$ at each authority $AA_{\theta }$. Even if the same user or organization performs the same operation again, it will obtain a different value. Therefore, the proposed scheme effectively prevents replay attacks.
- Man-in-the-Middle Attack: In the setup stage, KGC introduces a digital signature during the uniform random selection process of polynomial K(x). KGC signs each authority $AA_{\theta }$ with a hash function and sends the signature and K(x) together. Users also need to generate their digital signatures to prove their identity. During the user’s selection of $P_{\theta }$, they combine a temporary public key with their ID as their identity, sign identity, and integrate the signature with $P_{\theta }$ before sending it. Upon receiving the integrated data, the recipient performs corresponding verification calculations, such as the $hash^{\prime }$ function, to verify authenticity. If both identity authenticity and data integrity pass verification, this indicates no man-in-the-middle attack.
- Temporary Secret Leakage Attack: In defining the *n*-dimensional Gaussian function on lattice L(D), noise is introduced at each point. For example, when selecting parameter σ, a more significant parameter σ results in a smoother Gaussian function. A smooth Gaussian function helps improve the quality of noise. In the exponential function, the multiple different values involved in the calculation of ${\left\|x-c\right\|}^{2}$ result in noise. This noise interferes with adversaries when they attempt to analyze the trapdoor, meaning that the difficulty of temporary secret leakage is increased. Additionally, since the generation of ciphertext $E_{n}\left(M\right)$ use multiple parameters and multi-party computation, such as $c_{0}$, $c_{\theta }$, ${c^{+}}_{\theta ,i,1}$, ${c^{-}}_{\theta ,i,1}$, $c_{\theta ,i,1}$, and $c_{\theta ,i,2}$, in which each calculation is independent, temporary secret leakage of one user will not affect others. In other words, even if the ciphertext of one user is leaked, the ciphertext of other users should still be secure.
- Side-Channel Attack: In the RTrapGen algorithm, parameter σ is introduced and generated through a Gaussian distribution, so the generated threshold is random that can increase the difficulty for adversaries in analyzing the trapdoor. Adversaries cannot know the exact value of the trapdoor in advance. Furthermore, since the security of the algorithm relies on the assumption of RLWEs, it is difficult for adversaries to infer partial ciphertext from the encrypted trapdoor. The difficulty of the RLWEs problem is based on a theoretical mathematical problem that adversaries solve hardly within a finite time. Overall, the proposed scheme effectively prevents side-channel attacks.

The performance of the scheme is compared with those of other CP-ABE schemes. Through analysis of the number of authorities, system architecture, security, efficiency, and privacy protection, it is found that the proposed scheme outperforms the compared schemes in these aspects, as shown in Table 2.

The resistance to attacks is compared with references [5,8,10,13,20] in Table 3, which demonstrates their capabilities of addressing mainstream attacks in today’s network, traditional number theory cryptography, and quantum cryptography fields.

### 5.3. Performance Analysis

**Figure 2:**
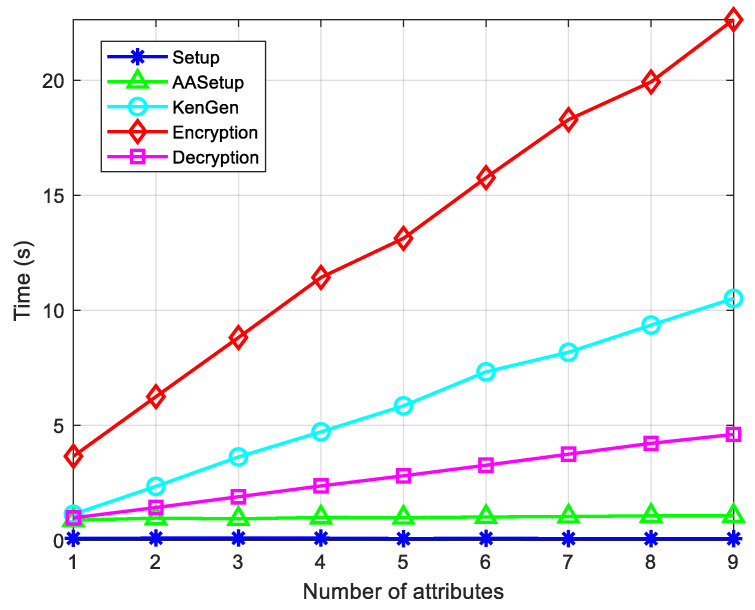
Time expenditure of each stage in the article.

We rely on the Ubuntu 20.04.6 LTS platform 12th Gen Intel^®^ Core™ i7-12700H × 12 64 bit version using Python 3.10 to simulate the proposed scheme. The experiment consists of five stages: (a) setup; (b) AASetup; (c) KeyGen; (d) encryption; (e) decryption. As shown in Figure 2, different user attributes are set to reflect the time expenditure of each stage.

**Figure 3:**
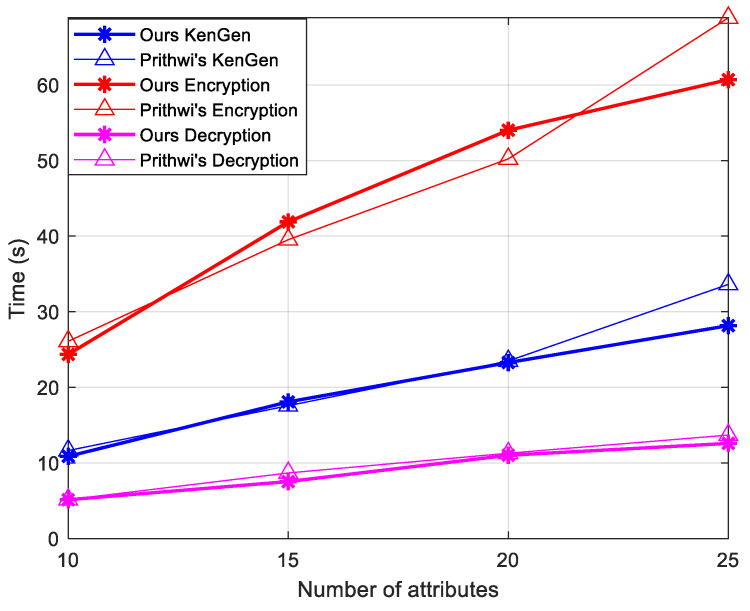
Time expenditure between different schemes in each stage.

As shown in Figure 3 and Table 4, compared with Prithwi et al. [5], the time gap between the two parties becomes more prominent as the number of user attributes increases, especially during the encryption and decryption processes. This further highlights the robustness of the proposed scheme in this paper. In Table 4, the computational costs of the various literature are compared, which leads to the conclusion that our protocol has more advantages. All experimental results represent the average time that the scheme run 20 times.

## 6. Summary

In this paper, an enhanced lattice-based post-quantum multi-authority CP-ABE and identity authentication scheme is presented based on the RLWEs problem. The deterministic standard deviation parameters in Gaussian sampling are adopted to improve algorithm efficiency compared with the original scheme. The scheme performance is optimized by judging different attribute sets, and the LLL algorithm resolves a set of scalar problems in linear algebra to reduce the computational cost of linear space checks. Due to the scheme support for multi-authority authorization, multi-authority authorization can be considered a distributed decentralized system. Compared with existing lattice-based CP-ABE algorithms, our improved scheme demonstrates higher efficiency.

## Figures and Tables

**Table 1:** Symbol meanings.

Symbol	Description
KGC	trusted key generation center
q	large prime number
h	total number of attributes in the system
$W^{\prime }$	total number of attributes in the access policy
$n_{k}$	number of attributes satisfying the access policy $n_{k}<h$
N	total number of authorities $AA_{\theta }$
f	degree of irreducible polynomial over a field of characteristic 2
P	generator(s) of group G
p	random large prime number(s) in number theory
η	belongs to a positive integer m=η|S|
$R_{q}$	Type $Z_{q}\left[x\right]/\langle x^{f}+1\rangle$ finite field, where $Z_{q}=\left\{0,1,\ldots ,q-1\right\}$, f are the highest degrees of polynomials, and q≡1(mod2f)
m	positive integer form $\left\lfloor \log_{b}q+1\right\rfloor +2$
$\left(b_{\theta ,i}^{+},b_{\theta ,i}^{-}\right)$	the public key portion used to generate $AA_{\theta }$.
$P_{\theta }$	the private key portion used to generate $AA_{\theta }$.

**Table 2:** Performance comparisons.

Scheme	Number of Authorities	Organization	Security	Efficiency	Privacy Protection
Reference [5]	Multi-authority	Distributed	High	High	High
Reference [8]	Single	Centralized	General	LOW	LOW
Reference [10]	Multi-authority	Distributed	General	General	General
Reference [13]	Multi-authority	Distributed	High	General	High
Reference [20]	Multi-authority	Distributed	General	High	General
This article	Multi-authority	Distributed	Higher	Higher	Higher

**Table 3:** Resistance to attacks. (√ represents yes, × represents no).

Scheme	Replay Attack	Man-in-the-Middle Attack	Temporary Secret Leakage Attack	Signal Leakage Attack
Reference [5]	√	×	√	√
Reference [8]	√	√	×	×
Reference [10]	√	√	√	×
Reference [13]	√	√	√	×
Reference [20]	√	√	√	×
This article	√	√	√	√

**Table 4:** Comparison of time costs for each proposal.

Scheme	Encryption Time Complexity	Decryption Time Complexity	Key Generation Time Complexity
Reference [5]	O((2mh+1+m)f⌈logq⌉)	O(hmf⌈logq⌉)	$O\left(\left(2h-\left|W^{\prime }\right|+1\right)mf\left\lceil \log q\right\rceil \right)$
Reference [8]	O((2mh+1+mN)f⌈logq⌉)	$O\left(n_{k}mfd\right)$	$O\left(\left(2h-\left|W^{\prime }\right|+N\right)(m+1)f\left\lceil \log q\right\rceil \right)$
Reference [10]	O((mf|S|+ηf)⌈logq⌉)	$O\left(2n_{k}mf\left\lceil \log q\right\rceil \right)$	O(2|J|mf⌈logq⌉)
Reference [13]	O((2mh+1+mN)f⌈logq⌉)	$O\left(2\left(2n_{u}+n_{v}-n_{r}\right)mf\left\lceil \log q\right\rceil \right)$	$O\left(2\left(n_{a}+n_{v}-n_{k}+1\right)(2m+1)f\left\lceil \log q\right\rceil \right)$
Reference [20]	O((2mh+1+mN)f⌈logq⌉)	$O\left(n_{k}mf\left\lceil \log q\right\rceil \right)$	$O\left(\left(2h-\left|W^{\prime }\right|+N\right)mf\left\lceil \log q\right\rceil \right)$
This article	O((2mh+2N)f⌈logq⌉)	O(mNf⌈logq⌉)	$O\left(\left(2h-\left|W^{\prime }\right|\right)mf\left\lceil \log q\right\rceil \right)$

## Data Availability

Data are contained within the article.
